# Nuclear Spin Relaxation of Longitudinal and Singlet Order in Liquid-CO_2_ Solutions

**DOI:** 10.3389/fchem.2021.668044

**Published:** 2021-04-26

**Authors:** Aliki Moysiadi, Francesco Giustiniano, Andrew M. R. Hall, Topaz A. A. Cartlidge, Lynda J. Brown, Giuseppe Pileio

**Affiliations:** School of Chemistry, University of Southampton, Southampton, United Kingdom

**Keywords:** singlet spin order, liquid-CO_2_, nuclear magnetic resonance, long-lived spin states, nuclear spin relaxation

## Abstract

Hyperpolarization techniques can enormously enhance the NMR signal thus allowing the exploitation of hyperpolarized substrates for *in-vivo* MRI applications. The short lifetime of hyperpolarized spin order poses significant limitations in such applications. Spin order storage can be prolonged through the use of long-lived spin states. Additionally, the storage of spin polarization–either in the form of longitudinal or singlet order–can be prolonged in low viscosity solutions. Here, we report the use of low viscosity liquid-CO_2_ solutions to store nuclear spin polarization in the form of longitudinal and singlet order for extended periods. Our results demonstrate that this storage time can be considerably sustained in liquid-CO_2_ solutions in comparison to other low viscosity solvents, opening up the possibility of new, exciting storage experiments in the future.

## Introduction

Molecules that contain an “isolated” spin-1/2 pair of nuclei, offer the possibility to prepare a form of spin order, namely, singlet spin order (Carravetta and Levitt, 2004; Carravetta et al., 2004; Pileio, 2020) with the fundamental property of being long-lived. This is due to the fact that singlet spin order decays at a much slower rate than the longitudinal spin order conventionally used in most NMR experiments. This form of order has already been used in a range of different applications including: high-sensitivity quantification of ligand binding (Salvi et al., 2012; Buratto et al., 2014); measurements of slow translational dynamics (Cavadini et al., 2005; Ahuja et al., 2009; Dumez et al., 2014; Pileio et al., 2015; Pileio and Ostrowska, 2017; Tourell et al., 2018); and long-lived molecular tags to preserve information over a long time (DeVience et al., 2013; Feng et al., 2013; Zhang et al., 2014, 2015, 2016; Dumez et al., 2015; Theis et al., 2016; Mamone and Glöggler, 2018; Saul et al., 2019; Tanner et al., 2019; Yang et al., 2019). There is great potential for exploitation of long-lived spin order in high impact applications and in combination with techniques such as PHIP (Bowers and Weitekamp, 1987), SABRE (Adams et al., 2009), and dissolution-DNP (Ardenkjaer-Larsen et al., 2003) as a vehicle to preserve spin hyperpolarization. In the important fields of *in-vivo* MRI and molecular imaging, it is crucial to achieve the signal enhancement provided by such techniques. The capacity to preserve such enhancement for very long time periods so as to allow quality controls, transport and injection into the patient offers an exciting step forward. Moreover, the possibility to preserve hyperpolarization for hour-long periods would allow delocalisation of the point-of-production (the hyperpolarization equipment) from the point-of-use (the NMR/MRI machine). This presents many advantages but perhaps the most important is that the point-of-use does not necessarily need to be equipped with hyperpolarizer instrumentation and have specially trained personnel (in the case of dissolution-DNP this is very costly).

Recent progress in this field exploits the use, at the hyperpolarization stage, of radical-containing porous matrices that allow the storage of hyperpolarized longitudinal order in the form of a frozen solid which also displays very long lifetimes (Gajan et al., 2014).

Another possible way to achieve this decentralization involves the exploitation of long-lived spin states. Indeed, long-lived spin order with record lifetimes of 70 min in degassed acetone-*d*_6_ solutions at 20°C and 0.4 T field (Stevanato et al., 2015) and of 108 min at 30°C and 0.25 T field (Hall et al., 2020) have been reported. However, in general, the conditions which maximize the lifetime may be different to the conditions required at usage. For example, storage in a low-viscosity solvent such as acetone can prolong the lifetime of those states, but such solvent is clearly incompatible for use in a clinical setting.

The rationale behind why the lifetime of spin order can be prolonged in low-viscosity media arises from the very core of nuclear spin relaxation theory (Kowalewski and Mäler, 2006). Spin relaxation is due to fluctuating magnetic fields present in solutions. These fields have a different nature and are ultimately due to spin-spin, spin-field and spin-rotation interactions. The contributions from different mechanisms are additive to the total relaxation rate. Moreover, the spin-spin mechanism due to dipole-dipole interactions between the two spins in the spin-1/2 pair (ipDD) dominates the relaxation rate in the case of longitudinal spin order in degassed samples. The ipDD mechanism, however, does not affect the singlet order lifetime (Levitt, 2012). The spin-field mechanism due to the chemical shift tensor anisotropy (CSA) is, typically, the second in order of importance and affects both longitudinal and singlet order. Spin-rotation mechanisms due either to the coupling between spin and angular moment (SR) or to the coupling between spin and internal motions (SIM) are the next in terms of importance; all other mechanisms including dipole-dipole interaction with out-of-pair spins, interactions with spins in solvent molecules and so on are of minor importance and can be neglected in a first approximation. A detailed discussion of these mechanisms and their role in singlet order relaxation has been summarized in a book chapter (Pileio, 2020).

The very fact that those magnetic fields fluctuate is due to the dynamics of molecules in solution including rotation, diffusion and collision events. The fundamental parameter used to characterize these fluctuations is the correlation time. The contribution to the total decay rate from ipDD and CSA mechanisms is proportional to a correlation time which is linked to the molecular rotational diffusion. This correlation time is usually indicated as τ_*c*_ but differentiated into τ_1_ and τ_2_ depending on the rank of the interaction (ipDD is a rank-2 interaction while CSA has rank-1 (CSA^−^) and rank-2 (CSA^+^) components). Both τ_1_ and τ_2_ are directly proportional to viscosity. Conversely, the contribution to the total decay rate from spin-rotation mechanisms is directly proportional to a correlation time which is linked to molecular collision. This correlation time is usually indicated as τ_*SR*_ but is inversely proportional to viscosity. This means that, depending on the relative strength of the active interactions, the longitudinal and singlet order lifetimes can be extended by reducing the viscosity of the solution. More appropriately, this lifetime extension is observed whenever the decay rates are dominated by mechanisms such as ipDD or CSA, whereas the opposite could be observed when SR mechanisms prevails.

As a consequence, we were interested in exploring the use of liquified CO_2_ gas as a low-viscosity solvent in which spin order can be stored either as longitudinal or singlet order. Pure liquid-CO_2_ has a viscosity of 0.06 cP which is significantly lower than, for example, pure acetone-*d*_6_ which has a viscosity of 0.34 cP, a factor of ~5.6 times lower. As previously outlined, in situations where ipDD and CSA relaxation mechanisms dominate, an elongation of singlet order lifetime is theoretically possible. A further significant advantage of exploiting liquid-CO_2_ is the ability to rapidly evaporate this solvent by simply venting the NMR tube, thus allowing easy exchange with another solvent. This would facilitate experiments that employ one set of conditions to prolong the storage of hyperpolarization and other conditions for the time of use.

The use of CO_2_ as a solvent in NMR is not new (Lamb et al., 1989; Bai et al., 1997; Gaemers et al., 1999; Gaemers and Elsevier, 2000; Yonker, 2000; Khodov et al., 2020) but our contribution is the first report in which the properties of this solvent are investigated in the context of enhancing the lifetime of long-lived spin order.

Herein, we describe equipment built to prepare and handle NMR tubes filled with liquid-CO_2_ solutions. We present a thorough investigation of the lifetime of longitudinal and singlet spin order in liquid-CO_2_ solutions as compared with the same values measured in more conventional organic solvents. We report data measured at a wide variety of magnetic fields from 16.4 T to 50 mT for three different molecular systems that all support long-lived spin order. These findings are discussed in terms of a simplified relaxation analysis, based on previously derived analytical equations, and we propose future experiments made possible by our results.

## Materials and Methods

### Instrumentation

Experiments presented in this paper were run on a variety of NMR instruments. Data at 16.4 T was collected on a Bruker 700 MHz Avance Neo spectrometer equipped with a 5 mm TCI prodigy cryoprobe. Data at 11.7 T was collected on a Bruker 500 MHz Avance III spectrometer equipped with a 5 mm TBO Z-gradient probe. Data at 7 T was collected on a Bruker 300 MHz Avance III spectrometer equipped with a Bruker MICWB40 microimaging probe carrying a ^1^H/^13^C 10 mm resonator. Data collected at magnetic fields below 7 T was collected in field-cycling mode by using an automatic sample shuttle (Hall et al., 2020) installed on the 300 MHz spectrometer.

### Molecular Systems

**Figure 1:**
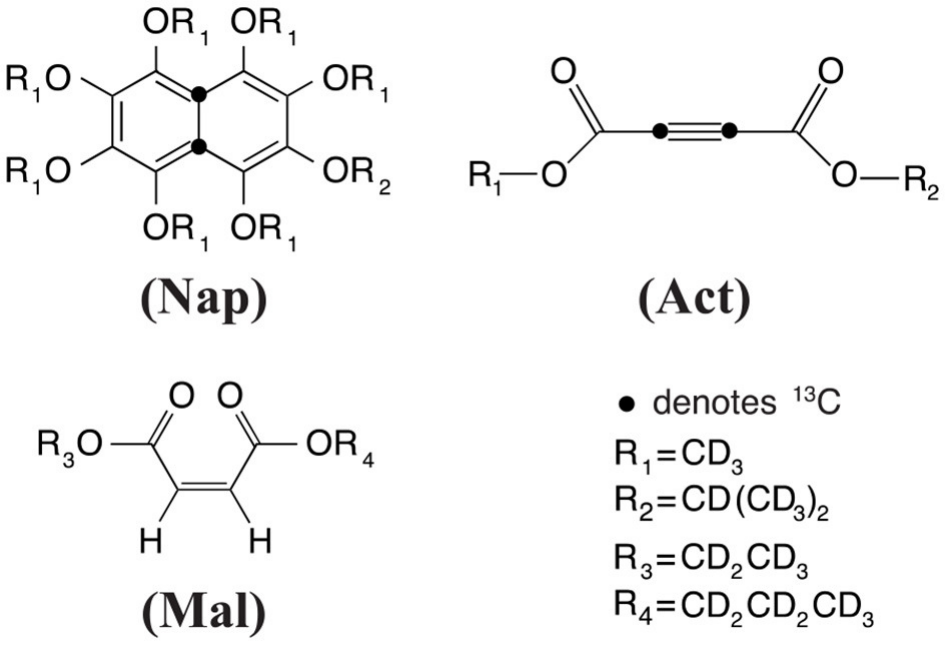
Structure of the molecular systems employed in this work.

Experiments have been carried out on a variety of molecular systems which support long-lived spin states. The molecular structures of all systems employed were reported in Figure 1. The first molecule is a doubly-^13^C-labeled and perdeuterated derivative of naphthalene, 1,2,3,4,5,6,8-heptakis(methoxy-*d*_3_)-7-((propan-2-yl-*d*_7_)oxy)-4a,8a-^13^C_2_-naphthalene (**Nap**). The second molecule is a doubly-^13^C-labeled and perdeuterated unsymmetrical diester of acetylene dicarboxylic acid, 1-(methyl-*d*_3_) 4-(propan-2-yl-*d*_7_) but-2-ynedioate (**Act**). The third molecule is a perdeuterated unsymmetrical diester of the maleic acid, 1-(ethyl-*d*_5_)-4-(propyl-*d*_7_)(*Z*)-but-2-enedioate (**Mal**). All molecules have been synthesized in-house according to published procedures (Pileio et al., 2012; Hill-Cousins et al., 2015; Brown, 2020).

### Samples

Molecules **Nap, Act**, and **Mal** have been used to prepare several different samples. For clarity we have labeled all preparations with different names and these are summarized in Table 1. The sample nomenclature works as follow: the first three digits reflect the molecular system; the next three digits refer to the solvent in which the molecule has been dissolved; the last digit, when present, distinguishes similar samples prepared at different concentration as detailed in Table 1.

**Table 1:** Nomenclature and preparation details of all samples used in the paper.

**Sample name**	**Molecular system**	**Solvent**	Viscosity of pure solvent at 20^**°**^C (cP)^*^	**Concentration (mM)**
**NapCo2a**	**Nap**	**liquid-CO**_**2**_ (**Co2**)	0.06	36
**NapCo2b**	**Nap**	**liquid-CO**_**2**_ (**Co2**)	0.06	61
**NapCo2c**	**Nap**	**liquid-CO**_**2**_ (**Co2**)	0.06	85
**NapCo2d**	**Nap**	**liquid-CO**_**2**_ (**Co2**)	0.06	108
**NapTbu**	**Nap**	**t-butanol-d**_**10**_ (**Tbu**)	4.3	200
**NapDms**	**Nap**	**DMSO-d**_**6**_ **(Dms)**	2.4	200
**NapEth**	**Nap**	**Ethanol-d**_**6**_ (**Eth**)	1.2	150
**NapClf**	**Nap**	**Chloroform-d** (**Clf**)	0.57	260
**NapMet**	**Nap**	**Methanol-d**_**4**_ (**Met**)	0.52	160
**NapAce**	**Nap**	**Acetone-d**_**6**_ (**Ace**)	0.34	200
**NapCo2e**	**Nap**	**liquid-CO**_**2**_ (**Co2**)	0.06	50
**ActClf**	**Act**	**Chloroform-d** (**Clf**)	0.57	700
**ActCo2**	**Act**	**liquid-CO**_**2**_ (**Co2**)	0.06	100
**MalEth**	**Mal**	**Ethanol-d**_**6**_ (**Eth**)	1.2	500
**MalCo2**	**Mal**	**liquid-CO**_**2**_ (**Co2**)	0.06	340

* *Handbuch der Instrumentellen Analytik NMR-spektroskopie provided by S. Thomas in “Spectroscopic Tools” URL: http://www.science-and-fun.de/tools/*.

Samples were degassed to remove paramagnetic dissolved oxygen. Excluding liquid-CO_2_ samples, degassing was done by ten freeze-pump-thaw cycles; the degassing procedure used for samples in liquid-CO_2_ is described below.

### High Pressure Tubes and Volume Restriction Inserts

**Figure 2:**
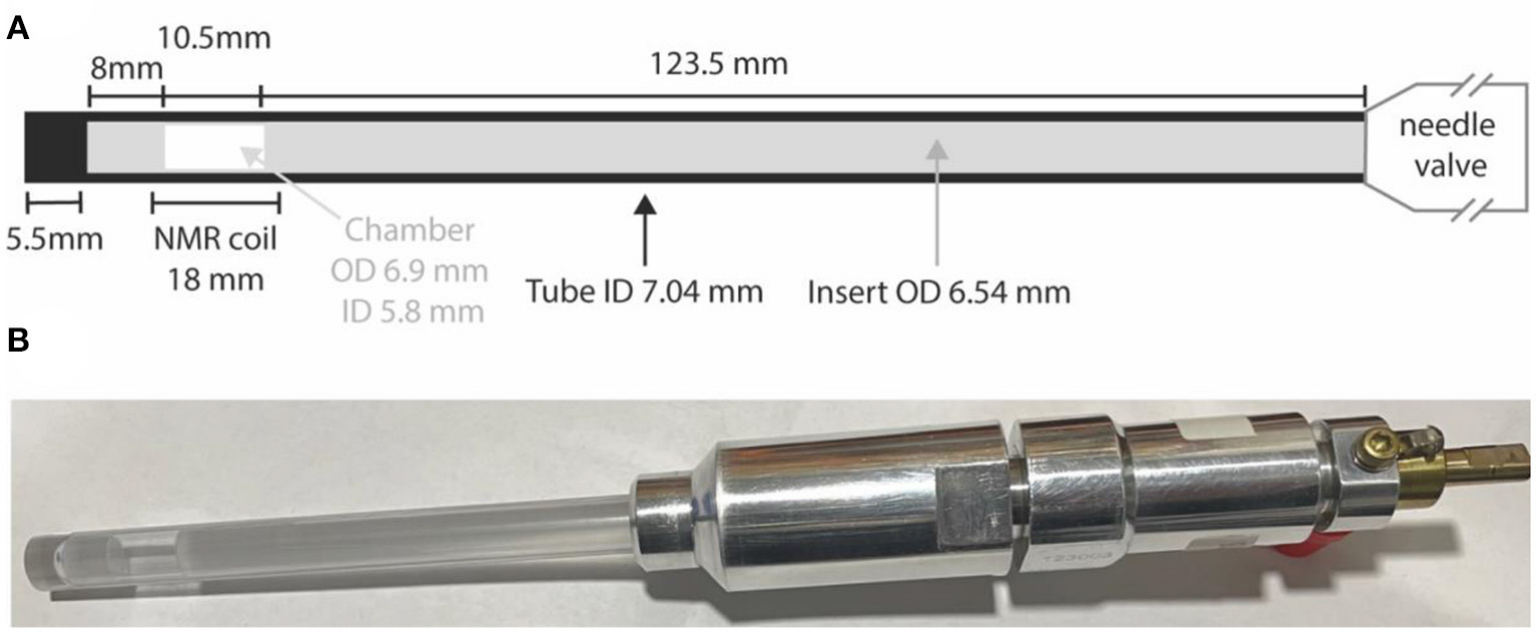
**(A)** A sketch of the high pressure NMR tube with details of the glass insert to restrict the sample volume; **(B)** a picture of the actual 10 mm sapphire high pressure tube with the glass insert and filled with a solution of **Nap** in liquid-CO_2_.

All samples other than those in liquid-CO_2_ have been prepared in standard 5 or 10 mm LPV NMR tubes. Samples involving liquid-CO_2_ are prepared in special high-pressure NMR tubes (purchased from Rototec-Spintec, DE) consisting of either a zirconia or sapphire tube connected to an aluminum needle-valve. High pressure tubes (5 mm) made of zirconia can withstand pressures up to 1,000 bar whilst 10 mm high pressure tubes are made of sapphire and can withstand pressures up to 300 bar. To confine the sample within the coil region (so as to minimize the effects of thermal convection) we constructed a glass insert consisting of two precision-manufactured filled glass rods and a piece of glass tube that fits inside the high-pressure tubes to confine the sample within a 10.5 mm cylindrical chamber placed in the middle of our 18 mm long coil. The insert outer diameter is slightly smaller than the high-pressure tube internal diameter (ID) leaving just a 250 μm gap. Tube construction and all dimensions are illustrated in Figure 2.

### CO_2_ Samples Preparation

**Figure 3:**
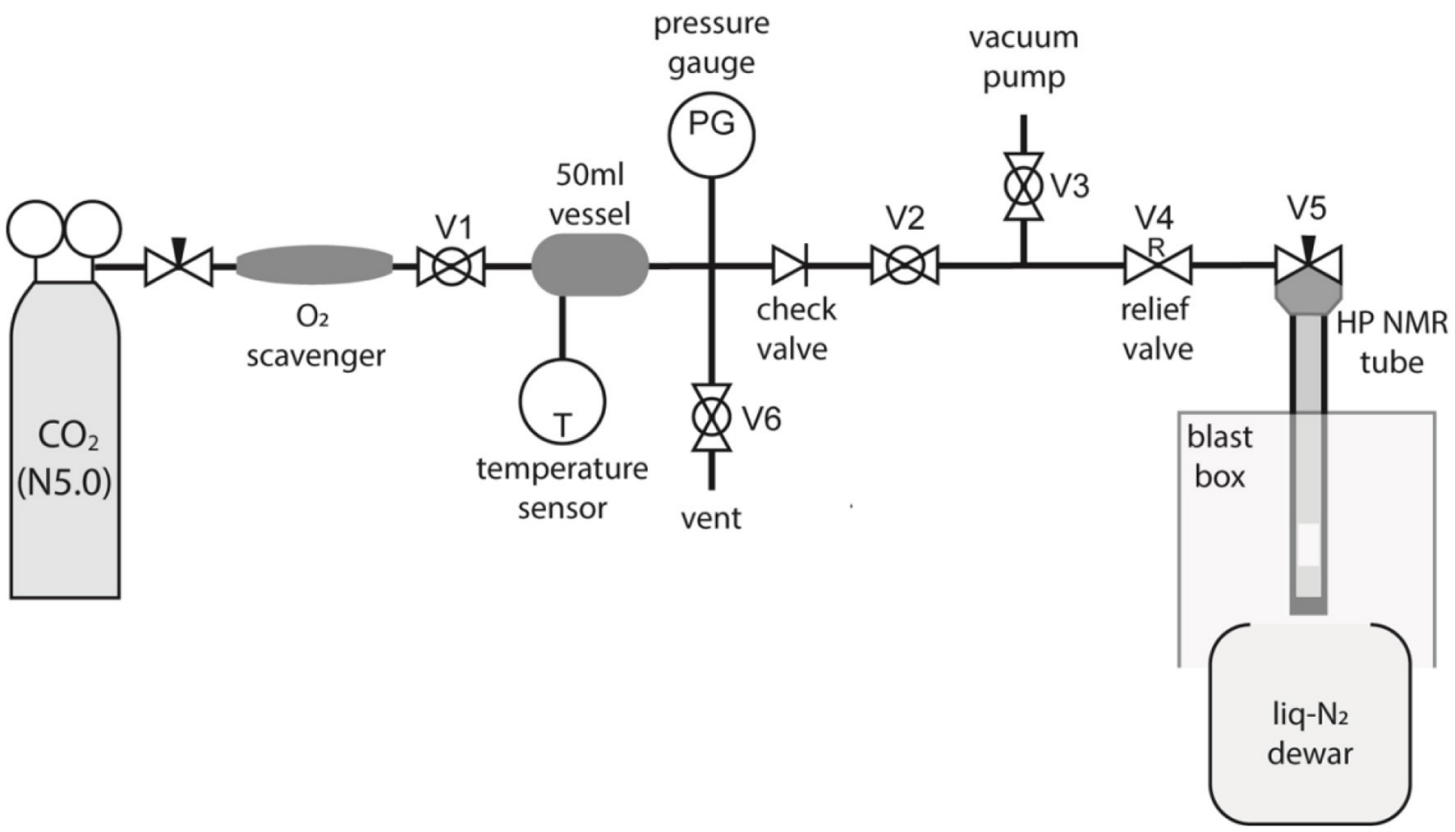
A diagram of the custom-made CO_2_ filling station built to fill high-pressure NMR tubes with liquid-CO_2_.

To allow preparation of samples in liquid-CO_2_ specialist equipment was required. The apparatus was constructed as diagrammatized in Figure 3. The “filling station” works by trapping a known amount of CO_2_ gas in a cylinder of known volume at room temperature and relatively low pressure, the amount required for a given experiment is then transferred into the high-pressure tube by cryogenic pumping using liquid-N_2_.

The gas from a N5.0 grade CO_2_ canister fills a 50 ml vessel at the desired pressure as monitored through a pressure sensor connected to the vessel. The vessel is at room temperature and the exact value of the temperature is measured and noted. The O_2_ content of the CO_2_ bottle has been measured to be 1 ppb using a OxyQC Wide Range oxygen meter by Anthon Paar; the gas was nevertheless filtered through a Restek high-capacity oxygen and moisture trap placed between the bottle and the 50 ml vessel to further remove O_2_. The whole tubing (including vessel and high-pressure tube) is filled with CO_2_ and evacuated a few times to remove the O_2_ possibly present in the equipment.

Successively, the desired amount of CO_2_ gas at the desired pressure is trapped in the 50 ml vessel, from where it is sucked into the high-pressure NMR tube (which already contains a given amount of the desired molecular system) by immersing the tube into the liquid-nitrogen Dewar. The CO_2_ gas liquifies or solidifies, depending on the exact conditions, inside the high-pressure tube and while under liquid-N_2_. Once the transfer occurs, the NMR tube needle valve is closed, and the tube is left to equilibrate at ambient temperature. The amount of gas needed to be trapped in the 50 ml vessel (*V*_*V*_ = 50 ml) is calculated on the basis of what amount of liquid-CO_2_ we want/need to fill the high-pressure tube with (Smith et al., 2013). In the preparations below where the high-pressure 10 mm sapphire tube is used, and similarly for the 5 mm zirconia case, our aim is to fill a 10.5 mm long and 5.8 mm ID chamber with liquid-CO_2_ (details in Figure 2). To do that we calculate the volume of liquid CO_2_ (*V*_*liq*_) as a function of the mass of CO_2_ (*M*_*C*_*O*__2__) to be trapped in that volume using:

(1) $$\begin{array}{l} V_{liq}=\frac{M_{CO_{2}}\;\chi_{liq}}{\rho_{liq}} \end{array}$$

where ρ_*liq*_ is the density of liquid-CO_2_ at the measured temperature (T) and the liquid fraction χ_*liq*_ is calculated once the density of liquid-CO_2_, the density of gas-CO_2_ (ρ_*gas*_) and the system density (ρ_*sys*_) are known:

(2) $$\begin{array}{l} \chi_{liq}=1-\frac{\rho_{gas}}{\rho_{sys}}\left(\frac{\rho_{liq}-\rho_{sys}}{{\rho_{liq}-\rho }_{gas}}\right) \end{array}$$

(3) $$\begin{array}{l} \rho_{sys}=\frac{M_{CO_{2}}}{V_{t}} \end{array}$$

*V*_*t*_ is the total free volume in the tube which is the sum of the free volume of the chamber sitting in the middle of the coil plus the free volume in the gap between the tube inner walls and the insert. Since T, ρ_*gas*_, ρ_*liq*_ and *V*_*t*_ are known we can set *V*_*liq*_ to match (or better to slightly exceed) the volume of the chamber placed in the NMR coil (*V*_*C*_) and therefore work out the mass of CO_2_ required. This mass is then calculated through the perfect gas law as:

(4) $$\begin{array}{l} M_{CO_{2}}=\frac{MW_{CO_{2}}\;P\;V_{V}}{R\;T} \end{array}$$

where *R* is the gas constant, *P* the pressure inside the vessel measured in our apparatus, and *MW*_*CO*_2__ the molecular weight of the gas. The required mass of CO_2_ is then dispensed by adjusting the pressure inside the 50 ml vessel. The value of the pressure for the preparation below typically ranges between 2 and 8 bars. For practical purposes it was advantageous to charge an additional two bars of CO_2_ (over the calculated value) into the 50 ml vessel. This ensured that when the required CO_2_ was removed from the vessel air was prevented from being drawn in, in the case of a leak, as the apparatus remained under two bars pressure.

As an illustrative example, to prepare sample **NapCo2e** in our 10 mm high-pressure sapphire tube with insert (see Figure 2) we have firstly calculated the tube free volume *V*_*t*_ = 1098 μl from known dimensions. Then, from tabulated values, we read ρ_*gas*_ = 0.1942 g/ml and ρ_*liq*_ = 0.7734 g/ml at the room temperature of 20°C. In this way, the mass of CO_2_ that can be trapped in the 50 ml vessel at a pressure of 4.8 bar is *M*_*CO*_2__ = 0.293 g which gives a ρ_*sys*_ = 0.394 g/ml. This value is below the critical value and therefore the NMR tube will contain a mixture of liquid and gas. The volume of the liquid is calculated from Equation 1 to be *V*_*liq*_ = 380 μl. Since the volume of the 10.5 mm chamber in the middle of the coil is *V*_*C*_ = 280 μl, then the amount of liquid-CO_2_ would fill the chamber and the gap above and below it for a few centimeters. To reach the concentration of 50 mM for this sample, we have inserted 7.9 mg of **Nap** (MW = 426.36 g mol^−1^). As discussed above we have therefore filled the 50 ml chamber with 6.8 bar of CO_2_ gas and then transferred the gas into the NMR tube until the pressure reading was two bars.

Once the sample equilibrates at room temperature (20°C in our case), the approximate pressure inside the NMR tube can be estimated from the pressure-density phase diagrams of pure CO_2_ (Smith et al., 2013) to be ~54 bars which is well within the tubes' tolerances (the value is only approximate because the phase diagram of our exact mixture is not available). Samples are moved around the laboratory within custom-made polycarbonate blast boxes and personnel wear face shields and gloves until the tube is safely placed into the probe.

### NMR Procedures

**Figure 4:**
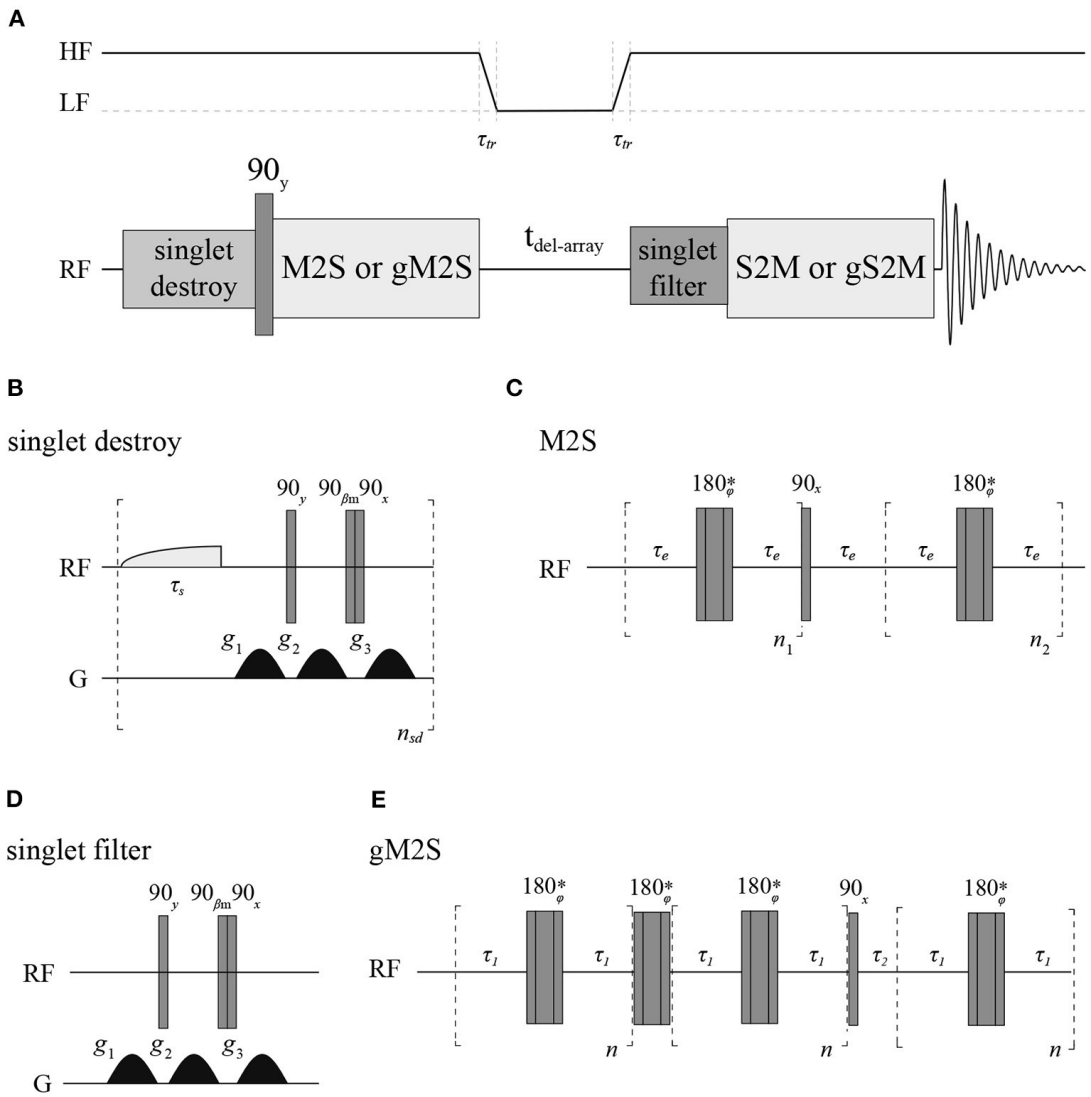
**(A)** Pulse sequence used to measure T_S_ with details of the singlet-destroy scheme **(B)**, M2S **(C)**, T_00_-filter **(D)**, and gM2S **(E)** blocks. The S2M and gS2M blocks are the time-reverse of M2S and gS2M, respectively. The asterisk indicates a composite 180° pulse built as 90x180y90x. The phase ϕ is cycled as [x,x,-x,-x,-x,x,x,- x,-x,-x,x,x,x,-x,-x,x] within the train of 180° pulses. All gradients have half-sinusoidal shape and β_*m*_ = *arctan(2*^1/2^*)*. The field variation indicated at the top of A is only used during the experiments run in field-cycling mode.

All longitudinal decay constants (T_1_) reported in this paper have been measured with a standard inversion recovery experiment. To measure singlet order decay constants (T_S_) we have used a sequence (Figure 4) where firstly any singlet order possibly present in the sample from the previous scan is destroyed (Rodin et al., 2019), singlet order is subsequently produced with either a M2S (Pileio et al., 2010) or gM2S (Bengs et al., 2020) pulse sequence, depending on the actual spin system features. The singlet order is then allowed to relax in a specific magnetic field for some variable delay time before being reconverted back into transverse magnetization by a S2M or gS2M and acquired. A singlet filter block is inserted before the S2M/gS2M to filter through only singlet order. All measurements at fields below 7 T were performed in a field-cycling mode using a custom-made sample shuttle (Hall et al., 2020). In these experiments the sample is (*i*) polarized in high field; (*ii*) magnetization inverted with a 180 degrees pulse (for T_1_) or converted to singlet order with a M2S or gM2S (for T_S_); (*iii*) sample is moved to a region of lower field along the magnet vertical stray field where longitudinal or singlet order are let to decay; (*iv*) after a variable amount of time the sample is shuttled back into high field where a 90 degrees pulse (for T_1_) or a S2M or gS2M are (for T_S_) is applied before signal detection.

The duration of a 90 degrees ^13^C pulse was 11.2, 25.0 and 27.5 μs at 16.4, 11.7 and 7 T, respectively, whereas the duration of the 90 degrees ^1^H pulse was 9.5 μs at 7 T. Typically, for ^13^C T_1_ and T_S_ experiments 8k points were collected using a 20 kHz spectral window. The recycling delay was fixed to 5T_1_. The number of transients was set to two for all T_1_ measurements and for T_1_ and T_S_ of **Mal**, **Act** and **Nap** in organic solvents whereas we have used four transients for T_S_ measurements of Act and Nap in liquid-CO_2_. The values of all parameters featuring in the pulse sequence of Figure 4 have been optimized around their theoretical values and the results are summarized in Table 2. The gradients featuring in the singlet filter are applied along the z-axis and have strength of 75, −75 and −75 mT m^−1^ and durations of 2.4, 1.4, and 1 ms, respectively. The singlet destroy scheme has been implemented using a qramp shaped pulse of duration τ_*s*_ = 1 s and maximum nutation frequency of 400 Hz. The sequence “shaped pulse-singlet filter” has been repeated n_sd_ = 5 times. In all field-cycling experiments the sample transport time τ_*tr*_ was set to 4 s.

**Table 2:** Experimental values of the pulse sequence parameters used for the various samples in measuring T_S_ with the pulse sequence in Figure 4.

**Sample**	**7 T**			**11.7 T**			**16.4 T**		
	**n_**1**_**	**n_**2**_**	**τ_*e*_(ms)**	**n_**1**_**	**n_**2**_**	**τ_*e*_(ms)**	**n_**1**_**	**n_**2**_**	**τ_*e*_(ms)**
NapTbu	14	7	4.6	8	4	4.6	6	3	4.6
NapDms	28	14	4.6	16	8	4.6	12	6	4.6
NapEth	16	8	4.6	10	5	4.6	6	3	4.6
NapClf	16	8	4.6	10	5	4.6	6	3	4.6
NapMet	16	8	4.6	10	5	4.6	6	3	4.6
NapAce	20	10	4.6	12	6	4.6	8	4	4.6
NapCo2e	22	11	4.6	12	6	4.6	8	4	4.6
MalEth	20	10	20.9						
MalCo2	44	22	20.9						
	**n**	**τ_1_(ms)**	**τ_2_(ms)**						
ActClf	3	1.10	0.7						
ActCo2	3	1.12	1.20						

## Experimental

### Naphthalene Derivative (Nap)

The naphthalene derivative (Hill-Cousins et al., 2015) (**Nap**) was chosen for initial investigations to develop and test the proposed procedures. The choice is based on the extraordinary long lifetime of the singlet order in this custom-designed and synthesized molecule (Stevanato et al., 2015; Hall et al., 2020).

#### Concentration Dependence of T_1_ and T_S_ in Liquid-CO_2_ Solutions

**Figure 5:**
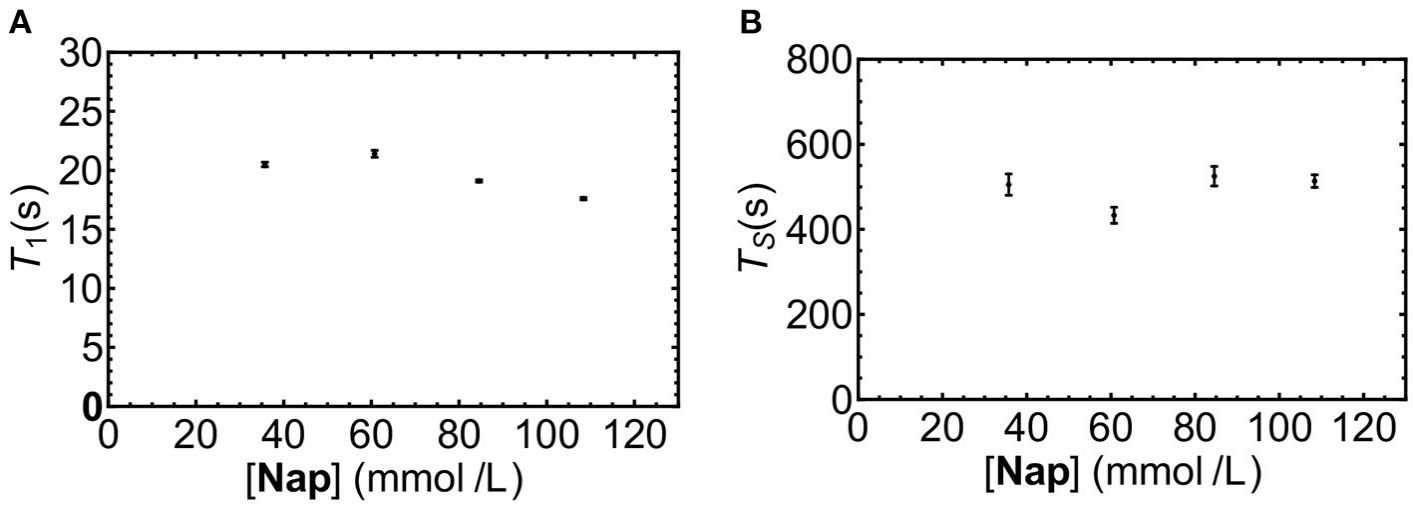
T_1_ **(A)** and T_S_ **(B)** for ***Nap*** dissolved in liquid-CO_2_ at different concentrations (samples ***NapCo2a-d***in Table 1).

The solubility of **Nap** in liquid-CO_2_ was not known, nor was it known if sample concentration would affect the observed decay time. As a preliminary investigation a series of solutions of increasing concentrations of **Nap** in liquid-CO_2_ at 20°C were prepared and T_1_ and T_S_ measured. Inspection of the results depicted in Figure 5 reveals no significant trend in T_1_ or T_S_ with increasing concentration, it was concluded that a working concentration of 50 mM **Nap** in liquid-CO_2_ was reasonable.

#### Viscosity Dependence of T_1_ and T_S_ in Liquid-CO_2_ Solutions

**Figure 6:**
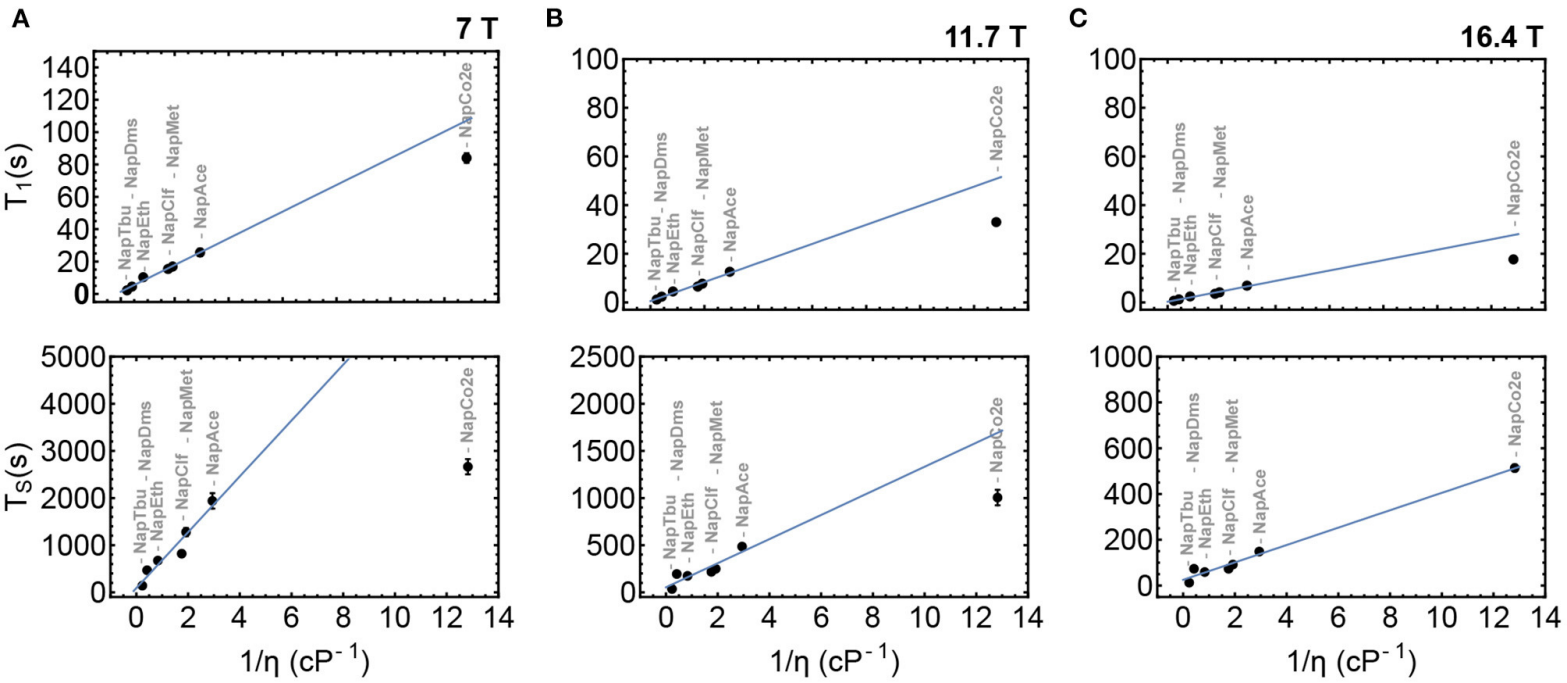
T_1_ and T_S_ as a function of inverse viscosity in samples ***NapTbu***-***NapCo2e***at 7, 11.7 and 16.4 Tesla fields in columns **(A–C)**, respectively. The blue line is the best fit to values for samples ***NapTbu***-***NapAce***.

To validate the initial hypothesis that both T_1_ and T_S_ can be prolonged in low-viscosity solutions, **Nap** was dissolved in a range of solvents of different viscosities from *tert*-butanol to liquid-CO_2_. T_1_ and T_S_ were measured in samples **NapTbu**, **NapDms**, **NapEth**, **NapClf**, **NapMet**, **NapAce**, and **NapCo2e** and results from these experiments are summarized in Figure 6. These measurements have been taken at three different magnetic fields: 7 T (Figure 6A), 11.7 T (Figure 6B) and 16.4 T (Figure 6C).

Both T_1_ and T_S_ correlate linearly with inverse viscosity in common organic solvents at all three field strengths. At 16.4 T, the values of T_1_ and T_S_ measured in liquid-CO_2_ solution (**NapCo2e**) also demonstrated a linear relationship with viscosity. Deviation from this behavior is observed at lower fields (Figures 6A,B) where the values of T_1_ and T_S_ for **NapCo2e** fail to meet the predicted value (blue line), indicating that, although the values of T_1_ and T_S_ are significantly increased in liquid-CO_2_, the time gain reduces as the magnetic field, at which relaxation occurs, reduces (Figure 6; Table 3).

**Table 3:** T_1_ and T_S_ values for samples ***NapEth***, ***NapAce*, **and ***NapCo2e***obtained at 20°C and different magnetic field strengths.

**Field (T)**	**T**_****1****_ **(s)**			**T**_****S****_ **(s)**		
	**NapEth**	**NapAce**	**NapCo2e**	**NapEth**	**NapAce**	**NapCo2e**
0.05	26 ± 2	72 ± 3	135 ± 26	1,350 ± 98	5,319 ± 682	4,553 ± 470
0.10	33 ± 5	75 ± 5	126 ± 18	1,587 ± 130	4,454 ± 369	^*^
0.25	25 ± 1	92 ± 11	176 ± 12	1,573 ± 70	4,460 ± 369	3,785 ± 318
0.5	30 ± 4	80 ± 5	158 ± 14	1,503 ± 52	5,146 ± 484	3,266 ± 192
1	32 ± 3	80 ± 8	148 ± 20	1,426 ± 76	5,075 ± 363	3,171 ± 153
2	30 ± 4	70 ± 10	175 ± 18	1,556 ± 21	4,429 ± 381	^*^
3	27 ± 1	57 ± 3	122 ± 7	1,291 ± 62	3,852 ± 302	2,851 ± 105
7	10 ± 1	30 ± 1	84 ± 3	620 ± 30	1,621 ± 208	2,664 ± 161
11.7	4.5 ± 0.1	16 ± 1	33 ± 1	174 ± 2	485 ± 16	1,005 ± 82
16.4	2.5 ± 0.1	7 ± 0.2	18 ± 0.1	59 ± 1	148 ± 5	513 ± 15

* *Data not collected*.

#### Field Dependence of T_1_ and T_S_ in Liquid-CO_2_ Solutions

The trends observed in Figure 6 are evidence for the following: at the highest field, the relaxation of singlet order is dominated by chemical shift anisotropy, a mechanism whose contribution to the relaxation decay constant is directly proportional to inverse viscosity; as the field is lowered, the contribution to the relaxation rate from other mechanisms prevails, such mechanisms would therefore have a different proportionality to viscosity.

If this is the case, further reducing the field to a value where the chemical shift anisotropy contribution becomes negligible would make the T_S_ in liquid-CO_2_ fall below any recorded values. The same is not expected for T_1_ as longitudinal order relaxation at any field would be dominated by the dipole-dipole mechanism whose contribution to the decay constant is inversely proportional to viscosity.

To investigate this hypothesis, we have measured the relaxation decay constants of longitudinal and singlet order at a range of fields between 50 mT and 7 T. Experiments were carried out in a field-cycling mode as described in Materials and Methods and for samples **NapEth, NapAce**, and **NapCo2e** where the labeled molecule **Nap** is dissolved in ethanol-*d*_6_, acetone-*d*_6_ and liquid-CO_2_, respectively.

Close examination of the results of the field-cycling experiments (Table 3) reveals that the T_1_ of all samples increases as the field is decreased (in all samples) indicating that the chemical shift anisotropy relaxation mechanism has a fundamental contribution to the observed decay constant at high field. However, at lower fields the T_1_ values in liquid-CO_2_ (**NapCo2e**) are significantly longer than those recorded in both ethanol-*d*_6_ (**NapEth**) and acetone-*d*_6_ (**NapAce**). The values of T_1_ measured in liquid-CO_2_ compared to ethanol-*d*_6_ are extended by a factor of ~7 at relatively high fields (16.4–7 T) but at lower fields this factor reduces to ~5. When considering the sample in acetone-*d*_6_ the extension factor is ~2 at all fields.

A similar trend can be seen for T_S_. The T_S_ in liquid-CO_2_ remains significantly longer than that measured in ethanol-*d*_6_ at all fields, whereas in acetone-*d*_6_, only at high fields the value of T_1_ is significantly longer in liquid CO_2_.

Interpreting our observations in a qualitative way, at high field, T_1_ is dominated mainly by the interplay of ipDD and CSA mechanisms, so the value of T_1_ increases as the CSA is progressively suppressed by transporting the sample to relax in a lower field. The contribution to the relaxation rate of both these mechanisms is expected to decrease as the viscosity reduces, explaining the significantly longer decay constants in liquid-CO_2_ with respect to ethanol-*d*_6_. The fall in lifetime extension of the liquid-CO_2_ sample in comparison to ethanol-*d*_6_ and acetone-*d*_6_ as the field is lowered is due to the presence of a mechanism whose contribution becomes more relevant once the CSA becomes of less importance. The explanation is similar for the T_S_ data, however, since singlet order is immune to the ipDD mechanism, at high field the singlet order relaxation is mainly dominated by CSA whilst, other mechanisms become more important at lower field.

### Acetylene Derivative (Act)

The field-cycling study to measure T_1_ and T_S_ as a function of magnetic field where relaxation occurs was repeated for the singlet-bearing acetylene derivative **Act** dissolved in CDCl_3_ (**ActClf**) and in liquid-CO_2_ (**ActCo2**) for comparison. Results from this study are summarized in Table 4.

**Table 4:** T_1_ and T_S_ values for samples ***ActClf***and ***ActCo2***obtained at 20°C and different magnetic field strengths.

**Field (T)**	**T**_****1****_ **(s)**		**T**_****S****_ **(s)**	
	**ActClf**	**ActCo2**	**ActClf**	**ActCo2**
0.05	34 ± 1	96 ± 2	4,123 ± 260	2,605 ± 258
0.10	32 ± 1	102 ± 9	3,675 ± 320	^*^
0.25	36 ± 2	127 ± 6	3,077 ± 300	^*^
0.5	37 ± 2	120 ± 5	2,914 ± 156	2,611 ± 82
1	41 ± 1	146 ± 12	1,468 ± 56	2,450 ± 120
3	47 ± 3	132 ± 8	214 ± 20	805 ± 78
5	35 ± 2	114 ± 6	100 ± 8	^*^
7	20 ± 1	78 ± 3	54 ± 6	218 ± 17

* *Data not collected*.

Again, both T_1_ and T_S_ increased as the field decreased, once more pointing toward a substantial role of CSA in the relaxation mechanism at high field. However, the T_1_ reaches a maximum at 3 T and then slowly diminishes again toward lower fields. There is a clear time gain of a factor of 3-4 approximately in longitudinal order lifetime when using liquid-CO_2_ as a solvent in comparison to CDCl_3_. Interestingly, T_S_ shows a significant gain of a factor of ~4 in liquid-CO_2_ (from 7 T down to 3 T) but this factor diminishes and even inverts at lower fields, with the T_S_ in CDCl_3_ being longer than that measured in liquid-CO_2_ in very low field.

### Maleate Derivative (Mal)

In a third set of field-cycling experiments the T_1_ and T_S_ of molecule **Mal** was measured at a range of magnetic fields both in ethanol-*d*_6_ (**MalEth**) and liquid-CO_2_ (**MalCo2**). Results from this study are summarized in Table 5. In this sample the singlet order is created in the proton spin-pair and protons have notoriously much smaller chemical shift tensors than carbons. For this reason, it is not expected that the CSA relaxation mechanism contributes significantly to the total relaxation decay at any magnetic field. Indeed, from the data in the table the values of both T_1_ and T_S_ in either sample do not vary significantly as the field is lowered. Comparing the results in liquid-CO_2_ with those in ethanol-*d*_6_, a gain by a factor ~4 is observed for T_1_ but there is a more modest gain factor of ~1.4 observed for T_S_.

**Table 5:** T_1_ and T_S_ values for samples ***MalEth***and ***MalCo2***obtained at 20°C and different magnetic field strengths.

**Field (T)**	**T**_****1****_ **(s)**		**T**_****S****_ **(s)**	
	**MalEth**	**MalCo2**	**MalEth**	**MalCo2**
0.05	9.6 ± 0.1	37.6 ± 0.4	237 ± 2	294 ± 8
0.10	9.5 ± 0.2	37.6 ± 0.3	233 ± 2	325 ± 20
0.25	9.7 ± 0.2	39.2 ± 0.4	251 ± 4	327 ± 15
0.5	9.7 ± 0.2	39.4 ± 0.5	249 ± 6	344 ± 22
1	9.8 ± 0.2	41.2 ± 0.3	250 ± 6	376 ± 21
3	9.5 ± 0.1	43.8 ± 0.5	246 ± 9	364 ± 15
5	9.8 ± 0.1	44.3 ± 0.8	237 ± 8	331 ± 15
7	9.7 ± 0.1	43.8 ± 1.6	242 ± 6	359 ± 27

## Discussion

The data presented above can be interpreted using a relaxation analysis based on previously derived analytical equations for the contribution of different mechanisms to the total relaxation rate of singlet spin order (Pileio, 2010, 2020). To do so, it is better to discuss in terms of decay rates R_1_ = 1/T_1_ and R_S_ = 1/T_S_ since the contributions of different relaxation mechanisms to the rate is additive. Additionally, we are going to use a simplified model where only the intrapair dipole-dipole (ipDD), chemical shift anisotropy (CSA) and the coherent chemical shift leak (CSL) mechanism are explicitly introduced. The remaining contribution to the total decay rate will be introduced as an unknown mechanism and its value retrieved through data fitting.

The equations for the decay rates due to the cited mechanism have been largely discussed in literature (Pileio, 2020) and are reported here for convenience:

(5) $$\begin{array}{r} R_{1}^{ipDD}=\frac{3}{2}b_{jk}^{2}\tau_{2};R_{S}^{ipDD}=0\\ R_{1}^{CSA^{+}}=\frac{1}{10}{\gamma^{2}B}_{0}^{2}\tau_{2}\left(\|\delta_{\text{j}}^{+}{\|}^{2}+\|\delta_{\text{k}}^{+}{\|}^{2}\right);\\ R_{S}^{CSA^{+}}=\frac{2}{9}{\gamma^{2}B}_{0}^{2}\tau_{2}\|\delta_{\text{j}}^{+}-\delta_{\text{k}}^{+}\;{\|}^{2}\\ R_{1}^{CSA^{-}}=\frac{1}{6}{\gamma^{2}B}_{0}^{2}\tau_{1}\left(\|\delta_{\text{j}}^{-}{\|}^{2}+\|\delta_{\text{k}}^{-}{\|}^{2}\right);\\ R_{S}^{CSA^{-}}=\frac{2}{9}{\gamma^{2}B}_{0}^{2}\tau_{1}\|\delta_{\text{j}}^{-}-\delta_{\text{k}}^{-}{\|}^{2}\\ R_{1}^{CSL}=0;R_{S}^{CSL}=\;\frac{{\gamma^{2}B}_{0}^{2}\Delta \delta_{iso}^{2}\tau_{2}b_{jk}^{2}}{12\pi^{2}J_{jk}^{2}} \end{array}$$

where $b_{jk}=-\hbar \mu_{0}\gamma^{2}/\left(4\pi r_{jk}^{3}\right)\;$ and *J*_*jk*_ are, respectively, the dipolar and indirect coupling constants between the two nuclei in the singlet spin-pair; *B*_0_ is the static magnetic field, Δδ_*iso*_ = δ_*j*_ − δ_*k*_ is the difference in the chemical shift of the two nuclei; τ_1_ and τ_2_ are the correlation times for rank-1 and rank-2 mechanisms, respectively and with τ_1_ = 3 τ_2_; γ is the gyromagnetic ratio; δ^+^and δ^−^ are the symmetric (+) and asymmetric (–) parts of the chemical shift tensor for the two nuclei in the singlet pair; ||δ|| indicates the Frobenius norm of the tensor δ.

The strategy adopted is based on the following assumptions:

- T_1_ in low field is dominated by the ipDD mechanism only, thus we can use $R_{1}^{ipDD}$ and the experimental value of T_1_ at 50 mT to retrieve the correlation time (other spin system parameters reported in Table 6)
  (6) $$\begin{array}{l} \tau_{2}=\frac{2}{3b_{jk}^{2}T_{1}\left(50mT\right)} \end{array}$$
- T_1_ at any other field is due to the effect of ipDD and CSA mechanisms:
  (7) $$\begin{array}{l} {\text{T}}_{1}=1/\left(R_{1}^{ipDD}+R_{1}^{CSA^{+}}+R_{1}^{CSA^{-}}\right) \end{array}$$
- T_S_ at all fields is due to the combination of CSA and CSL terms plus a further mechanism whose rate $R_{S}^{X}$ will be determined by fitting the experimental T_S_:
  (8) $$\begin{array}{l} T_{\text{S}}=1/\left(R_{S}^{CSA^{+}}+R_{S}^{CSA^{-}}+R_{S}^{CSL}+R_{S}^{X}\;\right) \end{array}$$

**Figure 7:**
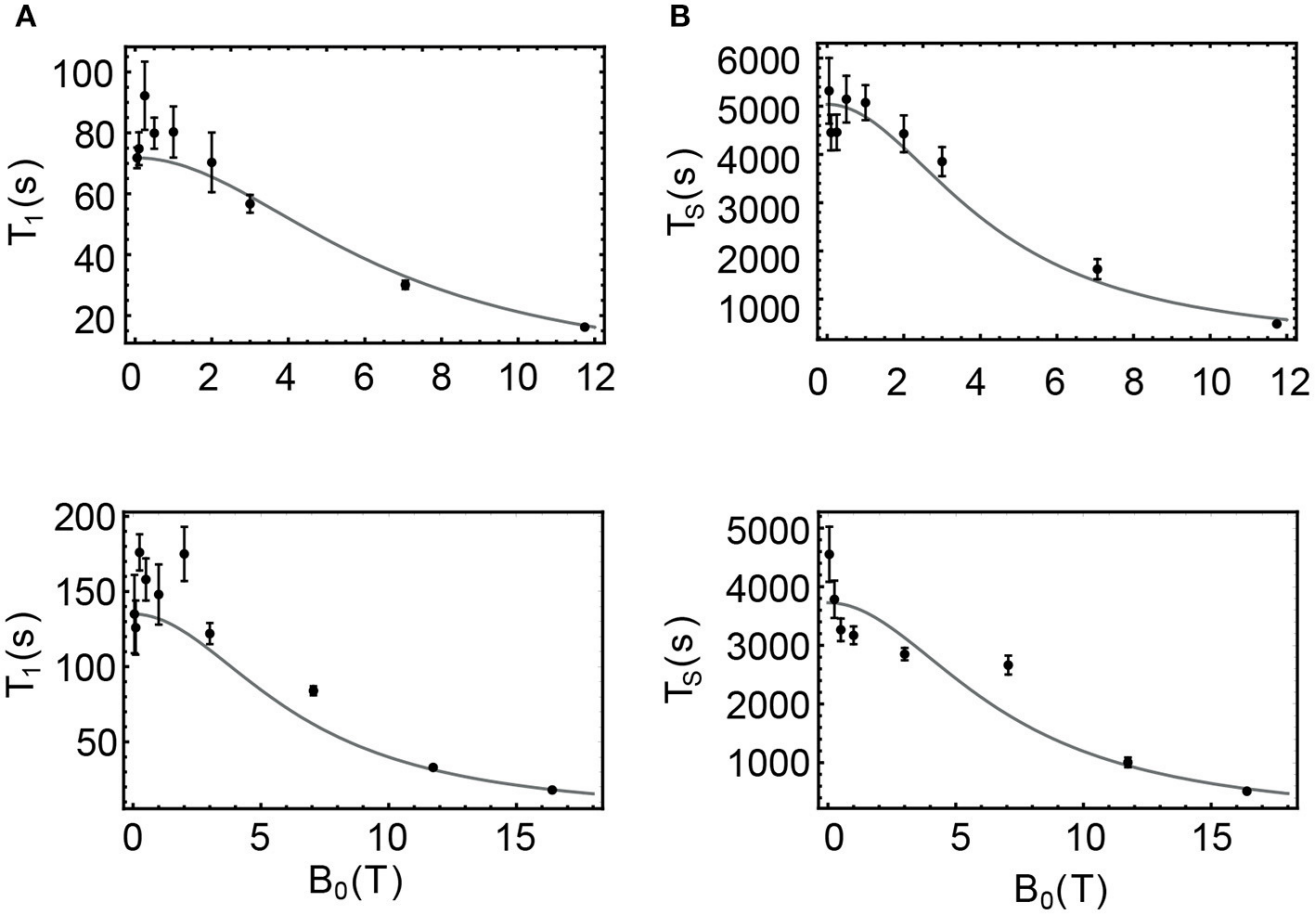
Filled circles are the values of T_1_ and T_S_ experimentally measured for samples ***NapAce* (A,B)** and ***NapCo2e* (C,D)**, also available in Table 3. The gray curves are the predicted values of these decay constants obtained using Equations 5–8.

In the case of sample **NapAce** and using Equation 6 we find a correlation time τ_2_ = 31 ps which can then be used to predict the value of T_1_ and T_S_ at any field. The values of T_1_ predicted using Equation 7 are plotted as a continuous line in Figure 7A and overlapped with the experimental point of Table 3 for an easy comparison. In the case of T_S_ we have fitted the experimental data against Equation 8 and for the unknown $R_{S}^{X}$ which was found to be 198.5 × 10^−6^ s^−1^. The fitting is shown in Figure 7B.

**Table 6:** Parameters used in the relaxation analysis for the case of ***NapAce***and ***NapCo2e***.

**Parameter**	**NapAce**	**NapCo2e**
*r*_*jk*_	1.395 Å	1.395 Å
*J*_*jk*_	54.8 Hz	54.8 Hz
Δδ_*iso*_	0.057 ppm	0.052 ppm
$\left\|\delta_{\text{j}}^{+}\right\|$	107 ppm	
$\left\|\delta_{\text{k}}^{+}\right\|$	112 ppm	
$\left\|\delta_{\text{j}}^{-}\right\|$	2.6 ppm	
$\left\|\delta_{\text{k}}^{-}\right\|$	8.1 ppm	
$\left\|\delta_{\text{j}}^{+}-\delta_{\text{k}}^{+}\right\|$	6.7 ppm	
$\left\|\delta_{\text{j}}^{-}-\delta_{\text{k}}^{-}\right\|$	9.9 ppm	

*Chemical shift tensors have been calculated in Stevanato et al. (2015) and here are assumed identical for both samples*.

The same procedure was used to predict the values of T_1_ and T_S_ for the sample of **Nap** in liquid-CO_2_ (**NapCo2e)**. In this case and using Equation 6 we found a value of the correlation time of τ_2_ = 16 ps and the fitted value of $R_{S}^{X}$ was found to be 268.3 × 10^−6^ s^−1^. The predicted values for this case are shown in Figures 7C,D for T_1_ and T_S_, respectively.

The results of this approximate relaxation analysis can be summarized as follows: in agreement with the initial hypothesis, the T_1_ of these samples is essentially defined by the ipDD and CSA relaxation terms since the predicted values matches well the experimental points; in the case of T_S_, relaxation in low fields is governed by a mechanism that contributes with a rate of 195.5 × 10^−6^ for the case of **Nap** in acetone-d_6_ and 268.3 × 10^−6^ for the case of **Nap** in liquid-CO_2_. This additional mechanism seems to have less dependence on viscosity than ipDD or CSA since its value is higher in the less viscous liquid-CO_2_ sample. One possible candidate is the spin-rotation mechanism whose dependence on the solvent viscosity is opposite to that of ipDD and CSA. Indeed, a variant of the spin-rotation mechanism, known as spin-internal motion (SIM), has already been proposed as an important relaxation mechanism for the singlet spin order of **Nap** in a previous study (Stevanato et al., 2015).

## Conclusion

In this study we have run a thorough investigation of the lifetime of both longitudinal and singlet order decay times of three different molecules in liquid-CO_2_ solutions in comparison with the values measured in more common organic solvents. The motivation behind this work was the concept that longitudinal and singlet order lifetimes could be extended in low viscosity compressed gases in comparison to solvents which are liquid at ordinary pressures and temperatures. Significantly, we have shown that liquid-CO_2_ allows an extension of lifetime of at least two-fold when compared with lifetime available in acetone-*d*_6_, one of the lowest viscosity solvents available. However, and depending on the relaxation mechanisms acting, such gain may not be able to prolong the absolute lifetime of singlet order since the latter hits a plateau where relaxation seems to be dominated by mechanisms that do not necessarily benefit from the lower viscosity of the liquid-CO_2_ solution.

Nevertheless, the possibility to store spin polarization (and hence hyperpolarization) for as long as 76 min, in the case of **NapCo2e**, at 50 mT, but in a solvent which can be removed almost instantaneously by simply opening the tube, suggests the possibility of new exciting experiments. Experiments where hyperpolarization is stored for tens of minutes in a substrate dissolved in liquid-CO_2_ and retrieved, at the time of use, by quick evaporation followed by dissolution in an experiment-compatible solvent which is perhaps not very convenient for hyperpolarization storage. We are currently building equipment to verify this hypothesis.

## Data Availability Statement

The original contributions presented in the study are included in the article/supplementary material, further inquiries can be directed to the corresponding author/s.

## Author Contributions

AM and TC ran experiments and processed data. FG built the CO_2_ equipment and provided engineering support. AH built the sample shuttle, ran experiments and processed data. LB synthesized the molecules and provided chemical support. GP devised the research, ran some experiments and wrote the paper. All authors contributed to the article and approved the submitted version.

## Conflict of Interest

The authors declare that the research was conducted in the absence of any commercial or financial relationships that could be construed as a potential conflict of interest.
